# A decade of evaluating burnout among American medical students using the OLBI-MS

**DOI:** 10.1038/s41598-025-22054-7

**Published:** 2026-09-06

**Authors:** Leah Teresa Rosen, Richard Friedman

**Affiliations:** 1 Weill Cornell Medical College, 1300 York Avenue, New York, NY 10021 USA; 2 Department of Psychiatry, Weill Cornell Medicine, New York, NY USA; 3 Present Address: University of California Los Angeles, Stein Eye Institute, Los Angeles, USA

**Keywords:** Human behaviour, Health care, Health occupations

## Abstract

Burnout among physicians is a growing concern, particularly during recent years in the setting of the Covid-19 pandemic. Recently, greater attention has been paid to researching physician burnout and its downstream effects (i.e. depression, suicidality); however, a majority of this research has focused on resident and attending physician populations. Medical students are a similarly vulnerable population, but the literature on medical student burnout is limited. Specifically, there is no existing literature that evaluates the trend of medical student burnout over recent years, particularly since the Covid-19 pandemic—a time when students navigated challenges related to their clinical experiences and virtual coursework. The aim of this study is to better understand the prevalence of feelings of burnout among American medical students, and evaluate the trend since the mid-2010s. Further, this study aims to compare average burnout scores among 2nd and 4th year medical students, to analyze the relationship between academic year and feelings of exhaustion and disengagement. We analyzed national data collected from the American Association of Medical Colleges’ Year Two and Graduation Questionnaire, each of which includes an Oldenburg Burnout Inventory for Medical Students. We found that since 2014, levels of reported exhaustion have significantly increased among medical students. We also found that between 2nd and 4th year students, the 4th year students have consistently reported lower levels of burnout than their 2nd year counterparts over time. Finally, we also found that burnout scores peaked among 2nd year medical students at the time of the Covid-19 pandemic, and have since trended down. These findings provide an essential understanding of burnout trends among American medical students, and can be utilized to help medical educators plan the timing and objectives of wellness interventions.

## Background

Physician burnout is a public health crisis that threatens the wellness of physicians, as well as the patients and communities for whom they care. Our definition of burnout stems from twentieth century psychological theories that describe the extent to which chronic workplace stressors are reflected in the psyche and spirit of the exposed workers, thus predisposing them to “crushing exhaustion, feelings of cynicism and alienation, and a sense of ineffectiveness—the trio known as burnout”^1^.

“Burnout” was officially added to the World Health Organization’s International Classification of Disease in 2019, and defined as an occupational syndrome emerging from chronic workplace stressors that have not been adequately addressed or managed^2^. The acceptance of this term by WHO speaks to the extent to which “burnout” has become ubiquitous.

That same year, a group of physicians within the Massachusetts Medical Society collaborated to publish a call to action on physician burnout. They stated firmly that “physician burnout is a public health crisis,” further explaining that “a primary impact of burnout is on physicians’ mental health, but it is clear that one can’t have a high performing health care system if physicians working within it are not well. Therefore, the true impact of burnout is the impact it will have on the health and well-being of the American public^3^”.

The call-to-action was a plea for institutions and society at large to recognize the extent to which the health of our communities is contingent on the health of our healthcare workers. Beyond outlining various concrete action steps for hospitals to take in order to mitigate physician burnout, the publication challenged readers to rethink their current approach to managing burnout. Specifically, they commented that wellness approaches—such as mindfulness, yoga, and resilience training—should only be deployed in conjunction with other systemic- and institutional-level reforms^3^. This multi-directional approach is informed by the understanding that burnout is not a personal problem, but a systemic one.

Jennifer Moss develops this idea in her 2019 article, “Burnout is about your workplace, not your people^4^.” She refers to an interview with Christina Maslach—developer of the original Burnout Inventory—where Maslach introduced the now popularized metaphor: a canary in a coal mine. Canaries were once used by coal miners to help detect the presence of toxic gases; the death of a canary thus indicated that the mine was not a safe work environment, not that there was something wrong with the canary^1^.

Maslach explains that when canaries enter a cave, “they are healthy birds, singing away…But when they come out full of soot and disease, no longer singing, can you imagine us asking why the canaries made themselves sick? No, because the answer would be obvious: the *coal mine* is making the birds sick^4^”.

The physician who experiences professional burnout can be viewed as a type of warning signal that the conditions of the workplace are unhealthy and even unsafe. And as the rate of physician burnout continues to rise across the United States, it is becoming even more clear that this is not a problem isolated to a handful of physicians at a few hospitals—*or a scattering of canaries within mines*. Rather, this is a plight so deeply ingrained within the system that it begs us to critically evaluate the entire practice of medicine—*or mining as a whole.*

One method of doing so is to focus on the experiences of medical students, with the goal of investigating the prevalence of burnout at this stage of training so to identify the optimal point of intervention. In a lecture titled “Doctors in Distress,” Dr. Ed Ellison remarks on the physician burnout crisis and prompts listeners to consider where the problem originates^5^. He then offers:

“Actually it begins in medical school, where we take these bright shining stars and we often wear them down to a dull nub. Studies show that students entering medical school actually have a higher incidence of wellness and resilience and optimism than those entering any other field of graduate study. And yet by the time they graduate, medical students’ sense of wellness and resilience and optimism is worse than that of any other graduate field.”

“So it makes us ask ourselves,” Dr. Ellison then continues, “what are we doing to our medical students?”^5^.

This particular question is one of utmost importance for several reasons. First, if medical school is indeed the starting point of physician burnout as Dr. Ellison theorizes, then it would follow that studying the specific experiences of American medical students would give us a clearer understanding of the specific factors that contribute to feelings of burnout. Further, the investigation of burnout among medical students also offers researchers the unique opportunity to devise interventions that can be placed upstream of the problem—that is, to mitigate physician burnout before medical students even become physicians. And finally, it is important to study the experience of medical students because there is a relative dearth of evidence in the literature about burnout among this community when compared to studies evaluating residents, fellows, and attending physicians^6^. 

Table 1 below summarizes the existing literature that specifically evaluates burnout longitudinally among American medical students^7–10^.

**Table 1:** A summary of the relevant literature on the subject of medical student burnout in the United States.

Study	Journal	Study type	Sample size	Conclusions
Dyrbye et al. (2008)	*Ann Intern Med*	Cross-sectional Study; Longitudinal Cohort Study	4287 (from 7 institutions)	- Burnout was reported by 49.6% of respondents - Suicidal ideation within the past year was reported by 11.2% of respondents - In the longitudinal cohort, burnout, quality of life, and depressive symptoms at baseline predicted suicidal ideation over the following year (P < 0.002 for all) - Burnout and low mental quality of life at baseline were independent predictors of suicidal ideation over the following year - Of the students who met criteria for burnout in the first year of the study, 26.8% had recovered by the second year - Recovery from burnout was associated with markedly less suicidal ideation
Dyrbye et al. (2010)	*Medical Education*	Longitudinal Cohort Study	792 (from five institutions)	Students who did not have burnout at either time-point (coined “resilient” students by the researcher) were less likely to experience depression, had a higher quality of life, were less likely to be employed, had experienced fewer stressful life events, reported higher levels of social support, perceived their learning climate more positively, and experienced less stress and fatigue (all *p* < 0.05) when compared to students who indicated burnout at either or both time points (“vulnerable” students) - The following factors were independently associated with recovery from burnout: perceiving student education as a priority for faculty/staff, experiencing less stress, not being employed, and being a minority - There were no differences in demographic characteristics between “resilient” and “vulnerable” students
Hansell et al. (2018)	*Fam Med*	Longitudinal Cohort Study (t = 4 years)	120 (within one medical school class)	- The overall presence of emotional exhaustion, depersonalization, stress, distress, and burnout significantly increased (p < 0.01) during the time between orientation and graduation - Distress and burnout scores both peaked at the end of Clerkship Year
Dyrbye et al. (2021)	*Academic Medicine*	Longitudinal Cohort Study (t = 4 years)	14,126 (national)	- Mistreatment reported during the second year of medical school was associated with higher exhaustion and disengagement scores, as well as higher odds of career regret (p < 0.0001) when measured at the time of graduation -A more positive emotional climate reported during the second year of medical school was associated with lower exhaustion and disengagement scores at the time of graduation

The main takeaways from these studies include the following:

1. Burnout was reported in nearly half of the medical students who responded to a 2008 survey—a rate of burnout comparable to physicians at the time^7,11^.
2. Suicidal ideation was reported in 1 out of 10 medical students who responded to a 2008 survey. Thus, suicidal ideation was more common among medical students than practicing physicians at the time^7,12^.
3. Rates of distress and burnout among students within one medical school class peaked at the end of their Clerkship year^9^.
4. A more positive emotional climate during the 2nd year of medical school was associated with lower rates of exhaustion and disengagement at the time of graduation^10^.

These research findings exemplify the urgency of the problem at hand. Not only are medical students in the US capable of experiencing burnout, they are already experiencing it at rates comparable to practicing physicians without yet bearing the full responsibility of patient care, paperwork, and administrative tasks. Further, medical students are also more likely to report suicidal ideation than practicing physicians^7,11,12^.

Dr. Liselotte Dyrbye has made substantial contributions to the literature and has in large part shaped our understanding of medical student burnout. Dr. Dyrbye’s research has demonstrated the utility of using longitudinal data to capture change in burnout scores throughout the course of medical school. Dr. Dyrbye has also pioneered the use of the Association of American Medical Colleges (AAMC) survey data within medical student burnout research^10^. Still, researchers have yet to explore the national trends in medical student burnout over an extended period of time (beyond 4 years), or compare the prevalence before and after the Covid-19 pandemic. It would be useful to evaluate these trends, as well as compare burnout scores at different times throughout medical education. This kind of research could help us understand if medical student burnout is a problem that has improved, worsened, or stayed the same over the past decade.

This study aims to evaluate the trend in burnout scores among American medical students over the past ten years, with the goal of ultimately identifying ways to improve circumstances for the future generation of physicians.

## Methods

At present, a robust way of assessing the well-being of US medical students on a national scale is to evaluate the AAMC survey data. The AAMC distributes multiple surveys to all Liaison Committee on Medical Education (LCME) accredited allopathic medical schools at various points throughout the curriculum. Two of these surveys—namely, the Year Two Questionnaire (Y2Q) and the Graduation Questionnaire (GQ)—feature an embedded Oldenburg Burnout Inventory (OLBI) modified for medical students (OLBI-MS)^13,14^.

The original OLBI is one of the tools developed to measure burnout, in addition to others including the various Maslach Burnout Inventories (MBI), the Copenhagen Burnout Inventory, and the Shirom-Melamed Burnout Measure. These many instruments were developed with different sample populations or definitions of burnout in mind. For instance, the MBI questionnaires were designed in accordance with the International Classification of Diseases definition of burnout as a workplace phenomenon resulting from “feelings of energy depletion or exhaustion; increased mental distance from one’s job, or feelings of negativism or cynicism related to one’s job; and reduced professional efficacy^2^.” Therefore, the MBI questionnaires focus on each of the above mentioned dimensions (exhaustion, disengagement, and professional inefficacy).

The development of the OLBI and other measures after the MLBI—the original and most widely distributed burnout assessment—reflects the challenging nature of defining or measuring burnout. Some researchers have theorized that the third dimension of the ICD definition—professional inefficacy—is not on its own a sign of burnout, but rather an effect of feelings of exhaustion or disengagement^15^. The OLBI, for example, is derived from the job demands-resources model of burnout. This model highlights the impact that workplace “demands” and “resources” have on an employee’s levels of exhaustion and engagement, respectively^16^. It excludes questions pertaining to professional efficacy.

There are, to that end, varying perspectives and definitions of burnout that drive the selection of a questionnaire. The AAMC chose to include the OLBI in their Y2Q and GQ surveys, and they modified it to substitute “work” with “medical school work.” Therefore, because the data to be presented here is derived entirely from the OLBI-MS, for the purposes of this paper we will define burnout as it is described by the creators of the OLBI in the job demands-resources model:

“The development of burnout follows two processes. In the first process, demanding aspects of work (i.e., extreme job demands) lead to constant overtaxing and in the end, exhaustion. In the second process, a lack of resources complicates the meeting of job demands, which further leads to withdrawal behavior. The long-term consequence of this withdrawal is disengagement from work”^16^.

The OLBI-MS consists of 16 items and measures two specific dimensions of burnout: disengagement and exhaustion. The disengagement subscale measures the extent to which a student reports feeling (1) distanced from course material or (2) negative attitudes toward school. The exhaustion subscale measures each student’s reported cognitive or physical strain due to the demands of medical school. Each subscale is scored from 0 to 24. Higher scores on this scale are correlated with higher levels of burnout^15^.

For the purposes of this study, we evaluated the Y2Q and GQ data from 2014 to present day. The OLBI-MS was first incorporated into the Y2Q in 2014, and the GQ in 2016. The Y2Q is distributed in October of second year and closes in January; the GQ is distributed in the second semester of fourth year. Respondent rates vary for each of these surveys and also vary by the year; moreover, an average of 12,000 individuals fill out each of the questionnaires every year.

To access these reports, we navigated to the “Data and Reports” section of the AAMC website. There, we searched for both the Y2Q and GQ, and then downloaded all available all schools summary reports. For the Y2Q, the earliest summary report spreadsheet on the AAMC website is from the year 2021, which also includes summary scores from 2019 and 2020 to trend the data. Similarly, the earliest GQ summary report spreadsheet available on the AAMC website is from 2022. To find the earlier data reports, we searched the given survey (Y2Q or GQ) and year into a search engine and were able to download the appropriate portable document formats (not spreadsheets). Of note, we requested a custom data report from the AAMC directly to receive access to de-identified individualized data reports, rather than the summary reports. Our aim was to evaluate the relationship between OLBI-MS scores and other factors including region of medical school, race, gender, and socioeconomic status. We first requested this custom data report in January 2023, then again in November 2023, and finally once more in February 2024. On all occasions, we were told that custom Y2Q or GQ data requests could not be provided “due (in part) to other outstanding projects and current bandwidth limitations.” Therefore, we were not able to access the individualized data reports and adjusted the aim of our project to focus on what was available: the summary data reports.

Our statistical analysis followed a standard comparison of means protocol to evaluate for a difference in burnout scores (1) between 2nd and 4th year medical students, (2) among 2nd year medical students over time, and (3) among 4th year medical students over time. This analysis involved calculating a pooled standard deviation, by using the standard deviations and sample sizes from two chosen comparison groups. Then, we conducted consecutive, two-sided t-tests, using MedCalc Statistical Software. We set the level of significance at *p* < 0.001.

## Results

### Disengagement subscale

Between the years 2016–2019, there was no significant difference in average disengagement subscale scores between 2nd year and 4th year medical students (*p* > 0.001). However, between 2020–2023, 2nd year medical students had significantly higher disengagement scores than 4th year medical students (t(df)_2020_ = -4.385, t(df)_2021_ = -15.118, t(df)_2022_ = -8.417, t(df)_2023_ = -4.147; *p* < 0.001 for all four years). The difference of highest magnitude (-0.7) occurred in 2021, when the 2nd year students reported an all-time peak average disengagement score (10.4 out of 24).

**Fig. 1:**
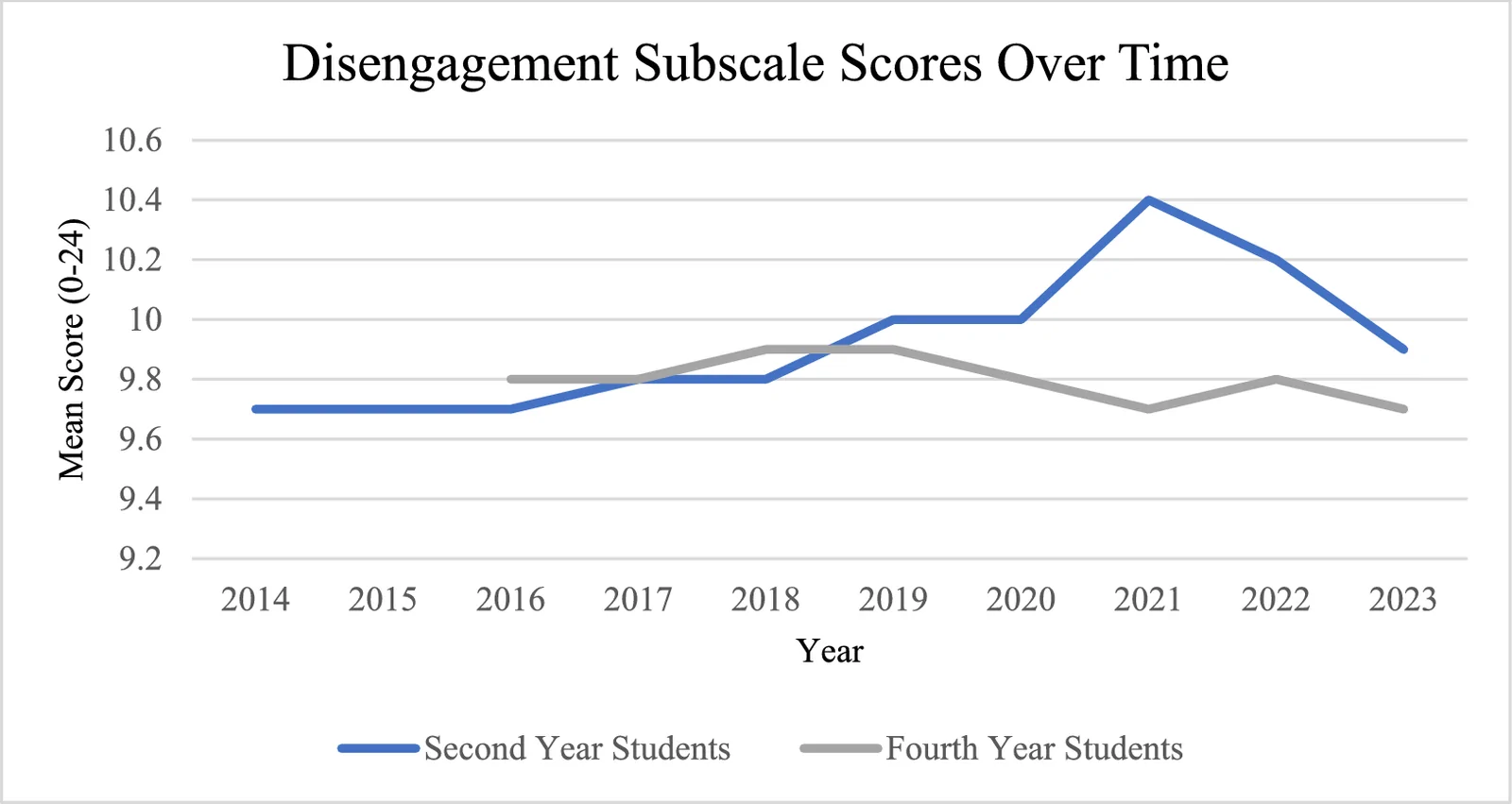
A depiction of average disengagement subscale scores reported by 2nd (blue) and 4th (grey) year medical students over time.

Over the course of 2014 to 2023, as depicted in Fig. 1, 2nd year medical students demonstrated a statistically significant rise in average disengagement scores (t(df) = 3.632; *p* < 0.001). Between 2016 and 2023, 4th year medical students reported no significant change in disengagement scores (*p* > 0.001).

### Exhaustion subscale

**Fig. 2:**
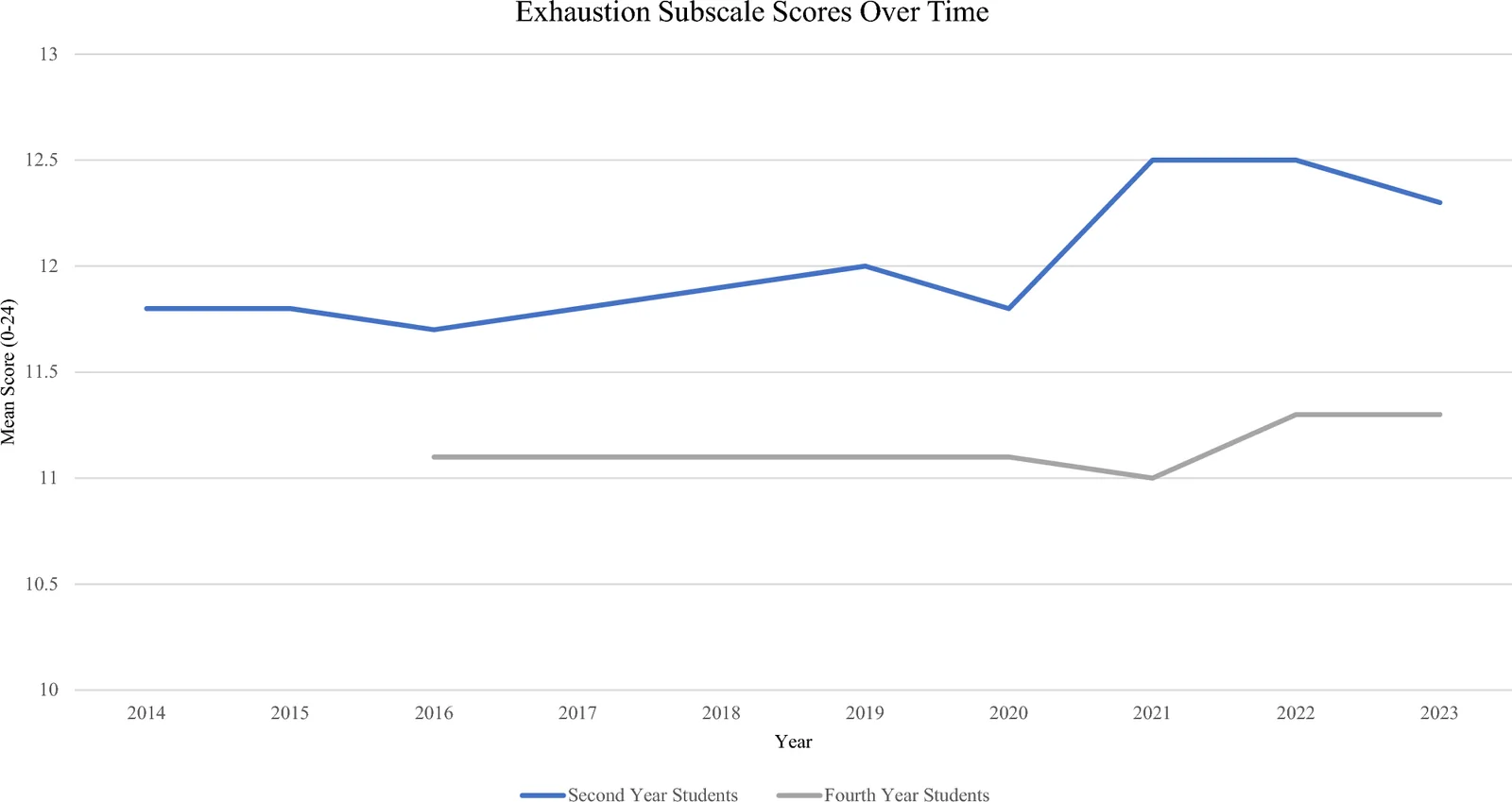
A depiction of average exhaustion subscale scores reported by 2nd (blue) and 4th (grey) year medical students over time.

As demonstrated in Fig. 2, 2nd year medical students reported a significantly higher average level of exhaustion than 4th year medical students between the years 2016–2023 (t(df)_2016_ = -12.717, t(df)_2017_ = -14.886, t(df)_2018_ = -17.744, t(df)_2019_ = -19.505, t(df)_2020_ = -14.767, t(df)_2021_ = -31.229, t(df)_2022_ = -24.761, t(df)_2023_ = -19.993; *p* < 0.001 for all eight years). The difference of highest magnitude between the two cohorts (-1.5 points) also occurred in 2021, when the 2nd year students reported an all-time peak average exhaustion score (12.5 out of 24 points).

Both cohorts—2nd and 4th year students—each demonstrated a statistically significant rise in exhaustion scores over the course of ten and eight years, respectively (t(df)_2nd years_ = 8.717, t(df)_4th years_ = 4.546; *p* < 0.001).

## Discussion

This study found that, on average, 2nd year medical students report higher burnout scores than 4th year medical students. This was surprising to us, as we had hypothesized that burnout scores would rise over the course of medical school as students gained more exposure to clinical work and assumed a greater sense of professional responsibility. It is important to note that the 4th year questionnaire is distributed in the spring semester, and thus the students fill out the OLBI-MS during the months surrounding Match Day and Graduation—times of relative reprieve, compared to clerkships, board exams, and residency applications^17^.

We also found that disengagement scores among 2nd year students have risen significantly over the past ten years, particularly in the setting of the Covid-19 pandemic. We had anticipated that burnout scores among medical students would rise during the pandemic—thus mirroring the exacerbation of burnout among practicing physicians that is well documented in the literature^18,19^. Moreover, we did not expect the magnitude of the spike to be as large as it was among 2nd year students, as illustrated in Fig. 1.

Additionally, we did not anticipate the relative drop in disengagement scores reported by 4th year students between the years 2019–2021 (t(df) = -4.744, *p* < 0.001). We suspect that the exacerbation of disengagement among 2nd year students and relative sparing of 4th year students during the Covid-19 pandemic is related to the different experiences that each of these cohorts had during that time. Again, medical students have limited required clinical responsibilities in the spring of their 4th year, while the growing adoption of accelerated pre-clinical medical curricula has given students increased exposure to the wards during their 2nd year^20,21^.

As depicted in Figs. 1 and 2, we found that the exhaustion subscale scores were notably higher than the disengagement subscale scores. This finding indicates that feelings of exhaustion may be a more considerable contributor to burnout among medical students, when compared to feelings of withdrawal or disengagement. In addition, exhaustion scores peaked among 2nd year students in the setting of the Covid-19—a pattern similar to that which was observed among disengagement scores. Interestingly, during the pandemic, the 2nd year students’ exhaustion scores declined before they spiked. In other words, the average exhaustion score among 2nd year students was significantly lower in the fall of 2020 than 2019 (t(df) = -3.979; *p* < 001). This reduction in exhaustion scores may be related to the shift towards virtual coursework that occurred at the time, but it is difficult to know with the use of quantitative data alone^22^.

We also found that, unlike disengagement scores, there was no overlap between 2nd year and 4th year exhaustion scores. The 2nd year students have reported feeling significantly more exhausted than the 4th year students for as long as OLBI-MS data has been collected from both cohorts (since 2016). Further, our findings suggest that the gap between exhaustion scores among 2nd and 4th year students has widened over time (Fig. 2).

Of note, the AAMC does not distribute the OLBI-MS to 1st or 3rd year medical students^13,14^. It is plausible that, based on the findings of this study, the burnout scores among 1st or 3rd year students could be higher than those reported by 2^nd^ year students, and by association, also those reported by 4th year students. The 3rd year of medical school is notoriously known to be grueling, as it traditionally includes the core clerkship curriculum as well as one or more board exams^23^. Further, some students find the 1st year of medical school to be significantly challenging, as it involves acclimating to a new environment and adjusting one’s study skills to fulfill academic expectations^24^. By not gathering data on burnout from these particular cohorts of students, we may be missing the peak of the problem.

## Limitations

Non-response bias is a common limitation to surveys gathering data about burnout and other well-being factors^25–27^. For instance, individuals who are experiencing the highest levels of burnout are less likely to have the emotional or physical bandwidth to fill out a survey—especially one as lengthy as the Y2Q (15–30 min) or GQ (30–45 min)^13,14,28^. Therefore, it is possible that the data collected by these AAMC surveys is skewed to under-represent the average burnout scores of 2nd and 4th year medical students.

Some medical schools also incentivize their students to complete these surveys by offering gift cards or raffle items. But to be entered into the raffle, students are asked to send evidence of completing the survey. By doing this, medical schools are compromising the anonymity of these surveys—a factor that protects the students. If students feel as if these surveys are not truly anonymous, they are less likely to complete the survey—and if they do, the accuracy of their responses is questionable as they may feel incentivized to answer at the behest of the medical school.

One limitation to the data is them being reported as average scores, rather than individualized numbers. If granted access to de-identified, individualized OLBI-MS scores, we could have evaluated the relationship between burnout and other factors such as gender, age, race, and region of medical school. We also could have used this data to trend burnout scores on an individual level, to evaluate how a given student’s scores differ at the two time points.

Another limitation to the data is the interpretation of the OLBI-MS scores. There is no accepted consensus regarding how the scores should be interpreted, and whether or not they can be organized into categories of low-medium–high cutoffs^29^. Further, a study aimed at assessing the validity of OLBI-MS concluded that the use of the inventory is not recommended to measure burnout among 2nd year medical students^15^. The generalizability of the 2nd year data is also limited, given that 2nd year curricular schedules vary significantly by medical school^30^.

## Conclusions

Overall, these are the takeaways from our study:

1. Using the OLBI-MS to measure two components of burnout (disengagement and exhaustion) in 2nd year medical students since 2014 and 4th year medical students since 2016, medical students in the United States report feeling more exhausted than disengaged.
2. Exhaustion and disengagement scores among 2nd year medical students both peaked in 2021, in the setting of the Covid-19 pandemic.
3. Exhaustion scores have risen significantly over time among 2nd year and 4th year medical students alike.
4. Since 2020, 4th year medical students have consistently reported lower levels of disengagement and exhaustion than their 2nd year counterparts.

These findings demonstrate the extent to which feelings of burnout among American medical students is a manifestation of exhaustion. Furthermore, to understand which particular factors are contributing to—or mitigating—these feelings of exhaustion, we need to re-evaluate which questions we ask and to whom. For one, while it is invaluable to have national-level survey data that utilizes a valid and reliable measure of burnout such as the OLBI, it is not clear whether the OLBI-MS is valid in its use among medical students^29^. The Maslach Burnout Inventory-Human Services Survey (MBI-HSS) for Medical Personnel, Single Item Burnout Measure, Copenhagen Burnout Inventory, Stanford Professional Fulfillment Index, and Well-Being Index are alternative questionnaires that are designed to measure burnout and other dimensions of well-being^31^. Many of these instruments offer insight into feelings beyond disengagement and exhaustion, which would provide researchers with a more comprehensive understanding of the experiences contributing to burnout among medical students.

Moreover, it would be highly valuable for the AAMC to also collect qualitative data from medical students about their experiences with burnout and other emotional challenges during medical school—similar to that which was accomplished through a free-text question in a recent study done in the United Kingdom^32^. To be able to read the anonymous, first-hand accounts of the medical students themselves would afford administrators, curriculum developers, and the medical education research community at large the opportunity to learn about the particular forces or experiences specific to given institutions that are negatively impacting our future generation of physicians. In theory, the AAMC could add two open-ended questions to their Y2Q and GQ, following the questions about burnout:

1. In your opinion, which factors contribute to feelings of burnout?
2. In your opinion, what changes would reduce feelings of burnout?

This proposed incorporation of qualitative data would grant the students the chance to articulate the complexities of their experiences, which survey scales and multiple choice questions cannot capture.

Further, the AAMC should distribute these questions to not only 2nd and 4th year medical students, but also 1st and 3rd year students. The inclusion of these school years would enable researchers to track trends in burnout scores throughout all four years of medical school. Particularly with the incorporation of qualitative responses, this change would aid in the identification of specific factors that contribute to—or protect students from—feelings of burnout. For instance, by gathering burnout scores and qualitative responses on an annual basis from medical students at every stage of their education, researchers and educators can then measure the relationship between curricular facets (i.e. pass-fail curriculum, clerkship grading, duration of pre-clerkship curriculum) and burnout.

Or, as an alternative to making changes to the current surveys—namely, which classes of students complete them, and which questions they include—we could more easily keep the surveys as they are, and rather expand our efforts to analyze and act upon their findings. The AAMC has collected years of important OLBI-MS data from the Y2Q and GQ, yet this data is difficult to access. The data that is accessible includes only summary score reports, from which researchers cannot study correlations among burnout scores and other factors.

For the purposes of this paper, the restrictions to data availability create a significant barrier to identifying trends in burnout or proposing solutions for medical students.

On a systemic level, reviewing data on burnout enables the AAMC and other organizations to measure the effects of broad changes (i.e. Step 1 becoming pass-fail, the Covid-19 pandemic) on medical student well-being. Further, the AAMC could establish an initiative to analyze these reports by medical school, with the intention of monitoring the effects of any changes implemented by the school: including revisions to the curriculum, learning environment, fatigue policies, or student housing. This undertaking would help the AAMC to identify any medical schools with particularly low burnout scores as exemplary models, as well as identify any schools with consistently high burnout scores or abrupt changes in their scores.

On an individual medical school level, we propose that administrators and deans use the OLBI-MS data to mitigate burnout within their school. Suggestions for how to do so include monitoring for changes in exhaustion and disengagement scores on an annual basis, as well as comparing the school’s scores to the national averages reported by the AAMC. If a medical school’s exhaustion scores are considerably higher, then the school can intervene by placing restrictions on working hours or offering protected study time during rotations. Similarly, if a school’s students are consistently reporting higher burnout scores than the national average, then that medical school should be responsible for developing a task force dedicated towards evaluating and addressing the issue.

Collecting the data on medical student burnout is an important first step, but we think that the AAMC has an obligation to share these data with both the medical students and their schools. The medical students have voluntarily provided the data to the AAMC and have a reasonable expectation that they will learn about the results, which bear directly on their emotional well-being. If students feel as if their feedback has not been honored or measurably incorporated into reformed practices within their medical schools, then they would likely be disincentivized from offering feedback in the future.

To the best of our knowledge, our study is the first of its kind, as most studies evaluating medical student burnout have only recruited within their institution^33–35^, or from others within their same state^36^. Other studies have utilized AAMC data, but have only looked at the results of one questionnaire rather than assessing for trends at different points of time during medical school^37–39^. The most similar large-scale study to utilize AAMC burnout data was done by Dyrbye et al. in 2021 but its main focus was not on burnout^40^; instead, it assessed the association between learning environment and subsequent outcomes (including burnout, empathy, and career regret). Our study has added to the existing literature by focusing solely on medical student burnout scores, expanding the time frame to look at up to ten years of data, highlighting the Covid-19 pandemic’s effect on medical student burnout scores, and proposing methods to improve our surveying of medical student burnout on a national level.

Our findings support the notion that medical students as a community warrant our attention and support, and we believe that the data presented represents an important step towards enacting meaningful change for these students. By investigating and addressing burnout at this early stage of training, we seek to improve the well-being of our future generation of physicians.

## Data Availability

The data analyzed for this manuscript is publicly available through the AAMC Data Reports (aamc.org/data-reports). Any additional data can be obtained from corresponding author on reasonable request.
